# Development of a Compendium of Energy Expenditures for Youth

**DOI:** 10.1186/1479-5868-5-45

**Published:** 2008-09-10

**Authors:** Kate Ridley, Barbara E Ainsworth, Tim S Olds

**Affiliations:** 1 Centre for the Analysis of Educational Futures, School of Education, Flinders University, Adelaide, Australia; 2 School of Applied Arts and Sciences, Department of Exercise and Wellness, Arizona State University, USA; 3 Sansom Institute, University of South Australia

## Abstract

**Background:**

This paper presents a Compendium of Energy Expenditures for use in scoring physical activity questionnaires and estimating energy expenditure levels in youth.

**Method/Results:**

Modeled after the adult Compendium of Physical Activities, the Compendium of Energy Expenditures for Youth contains a list of over 200 activities commonly performed by youth and their associated MET intensity levels. A review of existing data collected on the energy cost of youth performing activities was undertaken and incorporated into the compendium. About 35% of the activity MET levels were derived from energy cost data measured in youth and the remaining MET levels estimated from the adult compendium.

**Conclusion:**

The Compendium of Energy Expenditures for Youth is useful to researchers and practitioners interested in identifying physical activity and energy expenditure values in children and adolescents in a variety of settings.

## Background

In order to effectively explore relationships between physical activity (PA) and health, issues of measurement are critical. Assessment of energy balance associated with obesity and other metabolic health conditions relies on precise measurement of both total energy expenditure (EE) and energy intake (EI). Moreover, when comparing children's activity levels to PA guidelines, time spent in various intensities of activity (e.g. moderate to vigorous physical activity; MVPA ≥ 3 METs) must be estimated [1]. Currently researchers are using a wide variety of instruments to assess PA and energy expenditure for studies of health-related behavior in youth. These measures include objective measures, such as accelerometry and pedometry; and subjective measures, such as observation, proxy-report and self-report questionnaires. However measuring PA and EE [both resting metabolic rate (RMR) and activity related EE] in youth is difficult, no matter what instrument is used, particularly when attempting to define levels of sedentary, moderate and vigorous activity [2]. Therefore, researchers are constantly seeking to refine and improve the precision of their measures.

Whether objective or subjective measures of PA are used, in many cases, researchers refer to compendia of energy costs to supplement the data acquired from these measures when estimating EE [3]. Compendia may be used to assign energy costs to observational data when estimating total EE or time spent in varying intensities of activity. Energy costs from compendia may be assigned to activities identified as having been performed when a pedometer or accelerometer was removed. Compendia have also been used to evaluate accelerometer cut-points by comparing accelerometer counts to estimated MET-min cost (one MET-min is the energy required to sustain resting metabolism for one minute) of various activities [4]. However, the most common use of compendia is to convert self-report data into EE [5]. While much work has been done on developing compendia of energy costs for adults [3,6], there has been little research into the EE of youth performing everyday activities. The adult compendium contains only MET values measured in adults. While children's games are listed in the compendium, the energy cost is based on adults performing the games [7].

The use of adult data to assign energy costs to children's activities can be problematic. Energy cost per unit body weight tends to decrease as age increases [5,7]. Hence, it is widely agreed that using adult mass-specific ${\dot{\text{V}}\text{O}}_{2}$ data to assign energy costs to children and adolescents can result in substantial errors [3]. Two recent studies have investigated techniques used to assign energy costs to youth [8,9]. Ridley and Olds [9] undertook a review of data published on the energy costs of everyday activities performed by children and adolescents. Briefly, the review [9] evaluated four existing methods for assigning EEs to children, i.e. using adult METs and methods recommended Torun [10], Sallis and colleagues [5] and the FAO/WHO/UNU [11]. A literature search was conducted to locate all English language studies that measured energy costs in healthy 6.0–17.9 year olds using criterion EE measures. Combined datasets were created for walking (1187 data points), running (1974 data points) and all remaining activities (51 activities, 5592 data points). Comparative analyses (paired t-test, Bland Altman and intra-class coefficients) were used to compare the assigned MET cost values to the MET values calculated from measured EE data. Analyses revealed that using adult METs was the most accurate assignment technique of the four compared [9]. However, as the MET cost of both walking and running was significantly influenced by age, it was recommended that prediction equations based on age and speed be used to estimated MET costs of walking and running in children, rather than using adult METs [9].

Harrell and colleagues [8] measured the energy cost of 18 physical activities in 8–18 year olds. Although both studies were limited by insufficient data, in terms of the range of activities performed and a lack of subject diversity, both concluded that using adult METs, combined with child-specific RMRs, is the best existing technique to assign EEs when measured values are not available [8,9]. This recommendation suggests that although youth typically have higher RMRs than adults, resulting in a larger gross energy cost, the ratio of activity EE and resting EE appears to be similar in adults and youth [8,9]. The review of the energy costs of children and adolescents performing everyday activities [9] provided sufficient data to compile a compendium that includes MET values measured in youth where available. The purpose of this paper is to describe the development of a compendium of energy costs for youth (ages 6.0–17.9 y) and to provide the compendium for use by other researchers.

## Methods

### The six-digit activity code

Each activity within the compendium is assigned an individual code, loosely based on the system used by Ainsworth and colleagues [6]. The code consists of six digits which provide information about the characteristics of each activity. A description of the code structure is shown in Table 1. The six digit activity code is organized as follows. From the left, the first digit refers to the type of activity (1 = sedentary, 2 = transport, 3 = play/sport, 4 = school work, 5 = self care, 6 = chores, and 7 = other). The second digit refers to the body position while performing the activity (0 = sleeping, 1 = lying down, etc.) The third digit provides the context for the activity and is specific to each activity category (e.g. sedentary category: 0 = not attending to anything, 1 = watching TV, etc). The fourth and fifth digits describe the specific activity performed, and the sixth digit describes a self-rating of effort, for those activities that can be performed at varying intensities (0 = no self-rating of effort required; 1 = light, 2 = moderate, 3 = hard).

**Table 1:** The six-digit code used in the Compendium of Energy Expenditures for Youth.

**digit 1 ** **activity category**	**digit 2 ** **body position**	**digit 3 various**	**digits 4 & 5 specific activities**	**digit 6 self-perceived intensity**
**1 = sedentary**	0 = sleeping 1 = lying down 2 = sitting 3 = standing 4 = locomotion	0 = not attending to anything 1 = watching TV 2 = listening to music, radio 3 = reading 4 = conversing 5 = writing	individual activities numbered 00, 01, etc.	always 0

**2 = transport**	as above	0 = no equipment 1 = equipment	as above	0 = no self-perceived intensity required 1 = light 2 = moderate 3 = hard

**3 = play/sport**	as above	1 = individual activity 2 = partner/team activity	as above	as above

**4 = school work**	as above	always 0	as above	as above

**5 = self care**	as above	1 = bathroom activity 2 = eating 3 = dressing and undressing	as above	

**6 = chores**	as above	0 = food preparation 1 = tidying 2 = other 3 = garden	as above	as above

**7 = other**	as above	0 = musical instruments 1 = family, social, cultural activity 2 = other	as above	as above

*Note*: TV = television.

An example of a coded activity for talking on the phone – sitting (124100) is as follows (Table 2) :

**Table 2:** 

1	2	4	10	0
sedentary	lying down	conversing	activity number	no self-rating of effort required

Each activity is also assigned a MET level that can be used to score physical activity intensity levels for estimation of EE.

### Developing a list of activities

There are 244 activities listed in the compendium. These activities were chosen by scanning the adult compendium for activities likely to be undertaken by children, reviewing activity lists within existing physical activity questionnaires and reviewing papers that describe common activities performed by children [3,12]. The compendium for youth contains fewer activities than the adult compendium [3]. The adult compendium contains many activities that are not relevant for children and youth, e.g. occupational activities [3]. The youth compendium also contains fewer separate activity codes for variations in speed or intensity of movement. For example, 'walking' has over 30 variants in the adult compendium, including specifications such as variation in speed, terrain, etc; while the youth compendium only has six variants (i.e. walking – light effort; – moderate effort; – hard effort; and walking with a load – light effort; – moderate effort; – hard effort). It is unlikely that children can estimate their walking speed, thus the activity descriptions do not require that level of detail.

### Source of energy cost data

**Figure 1:**
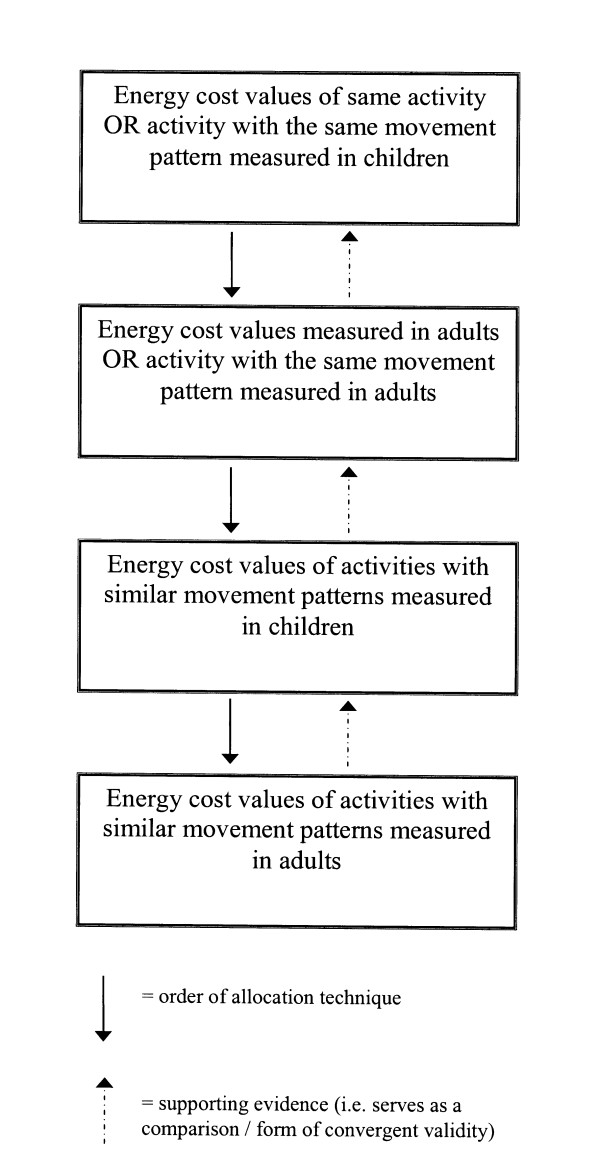
Hierarchy of energy cost value allocation decisions used in the Compendium of Energy Expenditures for Youth.

MET values were assigned to each activity based on the data located in the review of energy cost studies conducted by Ridley and Olds [9] and data sourced from the adult compendium. As the number of activities with measured child MET values is limited, a hierarchy of MET allocation techniques was used (outlined in Figure 1). The procedure involved evaluating whether activities had identical, or near identical, movement patterns to activities where energy costs had been measured in either children or were available in the adult compendium [3]. If the first allocation technique was not achievable due to a lack of data, the next technique was considered. Where data measured in youth were available from more than one study, a sample-weighted mean MET score was calculated [9]. Across the 244 activities, 35% of the MET values listed in the compendium were based on data measured in youth.

### Effort levels

Many of the activities selected for the compendium can be performed at a wide range of effort levels or intensities, e.g. games and sports. As many self-report questionnaires ask participants to self-rate their level of effort [13], items for each of these effort levels (e.g. volleyball – hard) have been added to the compendium for selected activities. Activities classified as < 3 METs were rarely assigned light, moderate and hard effort values due to the probable lack of variability in energy cost while performing these activities. Data were available for some activities to assist assignment of the MET costs to the various effort levels. For instance, data collected while "shooting hoops" was used to assign the basketball – light effort MET cost, while data collected while playing a game of basketball were used to assign the basketball – moderate effort MET cost. However in many instances measured MET values were not available separately for light, moderate and hard efforts. In order to devise an appropriate weighting strategy to apply to the mean measured MET value, those activities in the adult compendium with various intensities were investigated. For many activities the 'light' MET value was 0.75 × the 'moderate' value and the 'hard' value was 1.25 × 'moderate' value. These weightings were then compared to energy cost values measured in youth collated in the Ridley and Olds' review [9]. In many of the youth studies the weighted values of 0.75 and 1.25 corresponded to (approximately) the mean value ± 1 SD. Therefore, where only one mean measured MET value was available for an activity that could be performed at various effort levels, the mean MET value was multiplied by 0.75 and 1.25 respectively to assign 'light' and 'hard' values. For example, the 'moderate' MET value assigned for hopscotch is 5.9 based on two studies in children [14,15]. Based on the weighting strategy, the 'light' MET value for "hopscotch" was calculated as 4.4 (0.75 × 5.9) and the 'hard' MET value was calculated as 7.4 (1.25 × 5.9). While the decision to assign 'light' and 'hard' efforts for some activities by applying weightings of 0.75 and 1.25 was rather arbitrary, comparisons with existing data suggest this practice is reasonable given the lack of available data. As MET levels presented for each effort level are accompanied by a rationale for the calculation of the energy (see additional file 1: The Compendium of Energy Expenditures for Youth), researchers are able to make their own decisions, based on the characteristics of their data (e.g. availability of information regarding the amount of physical effort made while performing activities), whether to use the light- and vigorous-MET values allocated, or use the moderate value to assign the MET cost for all activities.

### Walking and running

Prediction equations were used to estimate walking and running MET costs. These equations were developed using level ground running and walking energy cost data from 40 studies collated in the Ridley and Olds review article [9]. The running MET prediction equation was based on 1974 data points: 0.27 age + 1.91 speed (m.s^-1^) + 0.46; r = 0.61, SEE = 1.38 METs. The walking MET prediction equation was based on 1187 data points: 0.07 age – 1.21 speed (m.s^-1^) + 1.65 speed^2 ^(m.s^-1^) + 1.72; r = 0.65, SEE = 1.0 MET. As previously mentioned, it was decided not to present numerous walking MET costs for specific speeds. Therefore, typical light, medium and hard speeds were used to derive the MET costs at these subjective intensities. The speeds chosen for light, medium and hard effort walking (0.97 m.s^-1^, 1.25 m.s^-1 ^and 1.53 m.s^-1^) and running (2.08 m.s^-1^, 2.50 m.s^-1 ^and 2.92 m.s^-1^) were selected based on speeds commonly reported in the Ridley and Olds energy cost review [9] and the customary slow, normal and fast walking speeds of 6–19 year olds reported in Waters and colleagues' standard tables [16]. Researchers using the compendium may choose to use the typical light, medium and hard MET costs for a 12 year old child, or if age and walk/run speeds are available, calculate MET costs using the prediction equations with age and speed (m.s^-1^) as inputs.

### Calculation of energy cost

In many research designs it is appropriate to analyse energy cost data in METs, without converting to kcal or kJ. For example, the calculation of physical activity level (PAL, a time-weighted mean MET score) or time spent in MVPA for comparison between groups, or across instruments, can be obtained without converting MET scores to another measure of EE. However, when research designs require a calculation of total or gross EE (e.g. comparison of total EE across groups, comparison of EE and energy intake within individuals, etc.), MET values from the compendium need to be multiplied by child RMRs (either measured or estimated): kcal = MET value × child RMR (kcal.kg^-1^.min^-1^) × kg body weight × number of minutes activity performed. Common prediction equations for child RMRs include Schofield's age-, gender-, and mass-specific prediction equations [17] and Harrell's age-, gender- and pubertal status-specific equations [8]. For example, the gross EE for a 45 kg child with an estimated RMR of 0.025 kcal.kg^-1^.min^-1 ^(estimated using Schofield's RMR equation [17]) performing 30 minutes of moderate basketball = 8.2 (MET) × 0.025 (RMR) × 45 (kg body wt) × 30 (minutes) = 276.75 kcal.

## Discussion and Limitations

The compendium was developed after a recent and extensive review of the published literature on the energy cost of activities in children and adolescents [9] and provides an up-to-date collation of currently available MET values for youth. In spite of this, due to a lack of energy cost studies, only 35% of the values listed in the compendium are based on data measured in youth, the rest are estimated from the adult compendium. The lack of data collected in youth is a limitation of the Compendium of Energy Expenditures for Youth. Nevertheless, evidence suggests that, on average, the magnitude of error is small when adult METs are used to estimate child MET costs and child RMRs are used as correction factors [9]. Moreover, despite the compendium replicating > 60% of its values from the adult compendium, the publication of a separate compendium of energy costs for youth is useful as it provides MET costs measured in youth where available and eliminates the need for researchers to locate and refer to numerous manuscripts to assign the most precise estimates of EE to their data. In addition, the youth compendium contains many activities commonly performed by children which are missing from the adult compendium (e.g. riding a scooter and playing playground games).

It is currently difficult, if not impossible; to eliminate error when assigning energy cost values to both adults and youth. As with many other measures of EE, the ability to precisely estimate EE using the compendium is limited. Both the adult and youth compendiums will more accurately estimate EE at a group level, rather than an individual level [18]. Many of the studies used to construct the compendium for youth had small sample sizes (e.g. < 20 subjects). A number were conducted many years ago and it is not clear whether the way children perform many activities, particularly leisure activities, has changed over the last few decades. Due to the high proportion of MET values sourced from the adult compendium, inherent limitations of the adult compendium may also be "passed on" to the youth compendium. A number of activities in the adult compendium were not directly measured, rather estimated based on the energy costs of other activities with similar movement patterns. Therefore the generalisability of the data in both the adult and youth compendiums may be questioned.

Using estimated mean MET values to assign energy costs to subjects is also problematic. In particular, activities that can be performed at varying intensities are most likely to have a wider variation in error of estimation. Not only are individuals able to perform the same activity at a range of intensities and varied level of mechanical efficiency, self-rated effort (or perceived level of intensity) can also impact on estimated EE. For example, two children could be performing the same physical activity, at the same rate of EE, yet one could rate the activity as of 'moderate' effort, while the other may perceive it as 'light'. Self-report instruments can be designed with features aimed at minimizing the extent of variation in self-rated effort [13]. Individuals may also vary in relation to weight status. While the use of METs to assign energy costs assumes the influence of body weight on energy cost is corrected for by applying a mass-specific RMR when estimating gross EE, it is unclear whether the MET costs of all activities, particular locomotor activities, are independent of body weight [19]. Therefore, some under- and over-estimation of energy cost related to the weight status of individuals may occur.

The limitations related to individual variation in movement, small sample sizes and paucity of data are not unique to the youth compendium. The adult compendium acknowledges the same limitations: "For activities in which the parameters are undefined, individual differences in EE can be large and the true energy cost for a person may or may not be close to the stated mean. This does not reduce the value of the standard intensity (MET) codes, but it is an important perspective from which to view the Compendium" [[6], pg. 73.]. Finally, the Compendium of Energy Expenditures for Youth is also not intended for use with children and adolescents who have disabilities that would significantly alter their movement patterns, mechanical efficiency and energy cost of activity.

## Conclusion

The Compendium of Energy Expenditures for Youth presented in the additional file 1 comprises 244 activities commonly performed by youth and associated MET costs. While the compendium will remain a work-in-progress with updated MET values being added to the compendium once data become available, the compendium provides the most up-to-date collation of energy costs for youth currently available. It is envisaged that the compendium will be of use to researchers investigating physical activity epidemiology in children and adolescents and will facilitate more accurate estimates of daily EE from subjective data.

## Notes

The authors have developed a database of energy cost data collected in children and adolescents to assist with future editions of the compendium. Researchers interested in sharing their unpublished or recently published data, or considering collecting energy cost data in the future, are encouraged to contact Dr. Kate Ridley for further information.

## Abbreviations

EE: energy expenditure; EI: energy intake; MET: metabolic equivalent; MVPA: moderate to vigorous physical activity; PA: physical activity; PAL: physical activity level; RMR: resting metabolic rate; SEE: standard error of estimate; TV: television.

## Competing interests

The authors declare that they have no competing interests.

## Authors' contributions

KR conceived the study, collated the energy cost data and designed the compendium. KR drafted the manuscript and made revisions at every stage. TO assisted in the design of the compendium, advised on the interpretation of the data and provided input at each stage of the manuscript draft. BA is an author of the adult compendium which provides the basis for the youth compendium. BA provided advice on the interpretation of the data and provided input at each stage of the manuscript draft. All authors read and approved the final manuscript.

## Supplementary Material

Additional file 1 The Compendium of Energy Expenditures for Youth. Table of activity descriptors and energy expenditure values. Click here for file
