# Supplementary material for: Development of a Compendium of Energy Expenditures for Youth
Source: Int J Behav Nutr Phys Act. 2008 Sep 10;5:45. doi: 10.1186/1479-5868-5-45 (PMC2564974; doi:10.1186/1479-5868-5-45)
Supplement: Additional file 1 — The Compendium of Energy Expenditures for Youth. Table of activity descriptors and energy expenditure values. [file 1479-5868-5-45-S1.pdf]

The Compendium of Energy Expenditures for Youth
(energy cost values derived from child measures are in bold)

Code	Activity	MET	Rationale for energy cost	Reference
	**SEDENTARY**			
**112020**	**listening to music/radio – lying**	**1.2**[a]	lying awake measured in 5 child studies, n = 130	[21-25]
**122160**	**listening to music/radio – sitting**	**1.3**	listening to radio, sitting measured in 1 child study, n = 22	[20]
**110150**	**lying awake**	**1.2**	lying awake measured in 5 child studies, n = 130	[21-25]
**113040**	**reading - lying down**	**1.2**[a]	lying awake measured in 5 child studies, n = 130	[21-25]
123110	reading - sitting	1.3[a]	Ainsworth 09030 - sitting - reading book, newspaper, etc	[3]
**114190**	**sending text messages (SMS) - lying down**	**1.2**[a]	lying awake measured in 5 child studies, n = 130	[21-25]
**124170**	**sending text messages (SMS) – sitting**	**1.5**	sitting playing game on a mobile phone measured in 1 child study, n = 19.	[26]
134180	sending text messages (SMS) - standing	1.8	Ainsworth 09050 - standing talking or talking on the phone	[3]
124090	sitting - talking	1.5	Ainsworth 09055 - sitting talking, or talking on the phone	[3]
**121130**	**sitting at the movies/cinema/theatre**	**1.2**[a]	sitting watching TV measured in 5 child studies, n = 470	[8, 33-36]
**120140**	**sitting quietly**	**1.4**	sitting quietly measured in 9 child studies, n = 288	[19, 21, 22, 25, 27-31]
100010	sleeping	0.9	Ainsworth 07030 - sleeping	[3]
134120	standing - talking	1.8	Ainsworth 09050 - standing talking or talking on the phone	[3]
**130060**	**standing quietly - e.g. standing in a line**	**1.5**	standing quietly measured in 6 child studies, n = 211	[19, 21, 22, 25, 27, 32]

**114070**	**talking on phone - lying down**	**1.2**[a]	lying awake measured in 5 child studies, n = 130	[21-25]
124100	talking on phone - sitting	1.5	Ainsworth 09055 - sitting talking, or talking on the phone	[3]
134170	talking on phone - standing	1.8	Ainsworth 09050 - standing talking or talking on the phone	[3]
**111030**	**watching TV - lying quietly**	**1.1**	watching TV lying down measured in 1 child study, n = 31	[24]
**121050**	**watching TV – sitting**	**1.2**	sitting watching TV measured in 5 child studies, n = 470	[8, 33-36]
**115080**	**writing - lying down**	**1.2**[a]	lying awake measured in 5 child studies, n = 130	[21-25]
	**TRANSPORT**			
**240070**	**climbing stairs**			
**240071**	**climbing stairs - light effort**	**5.3**	climbing stairs measured in 2 child studies, n = 224	[8, 35]
**240072**	**climbing stairs - moderate effort**	**7.0**	climbing stairs measured in 2 child studies, n = 224	[8, 35]
**240073**	**climbing stairs - hard effort**	**8.8**	climbing stairs measured in 2 child studies, n = 224	[8, 35]
241090	riding a motorcycle or motor scooter	2.5	Ainsworth 16030 - motor scooter, motorcycle	[3]
**221120**	**riding in a bus**	**1.4**[a]	sitting quietly measured in 9 child studies, n = 288	[19, 21, 22, 25, 27-31]
**221130**	**riding in a plane**	**1.4**[a]	sitting quietly measured in 9 child studies, n = 288	[19, 21, 22, 25, 27-31]
**221110**	**riding in a train/tram/ferry**	**1.4**[a]	sitting quietly measured in 9 child studies, n = 288	[19, 21, 22, 25, 27-31]
**221000**	**riding in/driving a car**	**1.4**[a]	sitting quietly measured in 9 child studies, n = 288	[19, 21, 22, 25, 27-31]
**240050**	**walking**			
**240051**	**walking - light effort**	**2.9**[b]	regression equation developed by Ridley and Olds: METs = 0.07 age – 1.21 speed ($m.s^{-1}$) + 1.65 speed^2 ($m.s^{-1}$) + 1.72, r = 0.65, SEE = 1.0 MET, n = 1187<br>Light speed[c] = 0.97 $m.s^{-1}$	[9]

**240052**	**walking - moderate effort**	**3.6[b]**	As above<br>Moderate speed[c] = 1.25 $m.s^{-1}$	[9]
**240053**	**walking - hard effort**	**4.6[b]**	As above<br>Hard speed[c] = 1.53 $m.s^{-1}$	[9]
**240090**	**walking carrying a load**			
**240091**	**walking carrying a load - light effort**	**3.4**	0.75 × moderate value	
**240092**	**walking carrying a load - moderate effort**	**4.2**	walking carrying an 8.5 kg backpack measured in 1 child study, n = 35	[32]
**240093**	**walking carrying a load - hard effort**	**5.3**	1.25 × moderate value	
241080	walking using crutches	4.0	Ainsworth 17140 - walking using crutches	[3]
	**PLAY/SPORT**			
341750	aerobics/health hustle			
341751	aerobics/health hustle - light effort	5.0	Ainsworth 03020 - aerobic, low impact	[3]
**341752**	**aerobics/health hustle - moderate effort**	**6.2**	aerobics (step) measured in 1 child study, n = 74	[35]
341753	aerobics/health hustle - hard effort	8.5	Ainsworth 03016 - aerobic, step	[3]
331000	archery	3.5	Ainsworth 15010 - archery (non-hunting)	[3]
341660	athletics (track and field): hurdles steeplechase			
341661	athletics (track and field): hurdles steeplechase - light effort	7.5	0.75 × moderate value	
341662	athletics (track and field): hurdles steeplechase - moderate effort	10.0	Ainsworth 15734 - track and field (steeplechase, hurdles)	[3]
341663	athletics (track and field): hurdles steeplechase - hard effort	12.5	1.25 × moderate value	
341650	athletics (track and field): jumping			
341651	athletics (track and field): jumping - light effort	4.5	0.75 × moderate value	

341652	athletics (track and field): jumping - moderate effort	6.0	Ainsworth 15733 - track and field (high, long, triple jump)	[3]
341653	athletics (track and field): jumping - hard effort	7.5	1.25 × moderate value	
341670	athletics (track and field): throwing			
341671	athletics (track and field): throwing - light effort	3.0	0.75 × moderate value	
341672	athletics (track and field): throwing - moderate effort	4.0	Ainsworth 15732 - track and field (shot, discus, hammer throw)	[3]
341673	athletics (track and field): throwing - hard effort	5.0	1.25 × moderate value	
342010	badminton			
342011	badminton - light effort	3.4	0.75 × moderate value	
342012	badminton - moderate effort	4.5	Ainsworth 15030 - badminton, general	[3]
342013	badminton - hard effort	5.6	1.25 × moderate value	
**341790**	**ballet**			
**341791**	**ballet - light effort**	**3.0**	0.75 × moderate value	
**341792**	**ballet - moderate effort**	**4.8**	dancing measured in one child study, n = 36	[27]
**341793**	**ballet - hard effort**	**7.0**	1.25 × moderate value	
342020	baseball			
342021	baseball - light effort	3.8	0.75 × moderate value	
342022	baseball - moderate effort	5.0	Ainsworth 15620 - baseball or softball, general	[3]
342023	baseball - hard effort	6.3	1.25 × moderate value	
**342030**	**basketball**			
**342031**	**basketball - light effort**	**7.2**	shooting hoops measured in 1 child study, n = 74	[35]
**342032**	**basketball - moderate effort**	**8.2**	basketball game measured in 1 child study, n = 12	[37]
**342033**	**basketball - hard effort**	**10.1**	basketball – fast dribbling and shooting measured in 1 child study, n = 19.	[26]

342050	bobsled toboggan luge			
342051	bobsled toboggan luge - light effort	5.3	0.75 × moderate value	
342052	bobsled toboggan luge - moderate effort	7.0	Ainsworth 19180 - sledding, tobogganing, bobsledding, luge	[3]
342053	bobsled toboggan luge - hard effort	8.8	1.25 × moderate value	
342070	broomball/floorball			
342071	broomball/floorball - light effort	5.3	0.75 × moderate value	
342072	broomball/floorball - moderate effort	7.0	Ainsworth 15130 - broomball	[3]
342073	broomball/floorball - hard effort	8.8	1.25 × moderate value	
341080	calisthenics			
341081	calisthenics - light effort	3.5	Ainsworth 02030 - calisthenics, light effort	[3]
341082	calisthenics - moderate effort	5.8	mean of light and hard values.	
341083	calisthenics - hard effort	8.0	Ainsworth 02020 - calisthenics, vigorous effort	[3]
**342850**	**chasey/tag/tips/tiggy**			
**342851**	**chasey/tag/tips/tiggy - light effort**	**3.8**	0.75 × moderate value	
**342852**	**chasey/tag/tips/tiggy - moderate effort**	**5.0**	playground games measured in 1 child study, n = 37	[27]
**342853**	**chasey/tag/tips/tiggy - hard effort**	**6.3**	1.25 × moderate value	
341840	climbing trees	8.0	movement pattern similar to rock climbing - Ainsworth 17120	[3]
**342100**	**cricket**			
**342101**	**cricket - light effort**	**2.6**	0.75 × moderate value	
**342102**	**cricket - moderate effort**	**3.5**	cricket measured in 1 child study, n = 35	[27]
**342103**	**cricket - hard effort**	**4.4**	1.25 × moderate value	
341110	croquet	2.5	Ainsworth 15160	[3]
342120	curling			

342121	curling - light effort	3.0	0.75 × moderate value	
342122	curling - moderate effort	4.0	Ainsworth 15170 - curling	[3]
342123	curling - hard effort	5.0	1.25 × moderate value	
**341130**	**dancing (general)**			
**341131**	**dancing (general) - light effort**	**4.1**	0.75 × moderate value	
**341132**	**dancing (general) - moderate effort**	**5.5**	dancing measured in one child study, n = 36	[27]
**341133**	**dancing (general) - hard effort**	**6.9**	1.25 × moderate value	
**342910**	**dodge ball/poison ball/brandy/speed ball**			
**342911**	**dodge ball/poison ball/brandy/speed ball - light effort**	**3.8**	0.75 × moderate value	
**342912**	**dodge ball/poison ball/brandy/speed ball - moderate effort**	**5.0**	playground games measured in 1 child study, n = 37	[27]
**342913**	**dodge ball/poison ball/brandy/speed ball - hard effort**	**6.3**	1.25 × moderate value	
342250	European handball (team)			
342251	European handball (team) - light effort	6.0	0.75 × moderate value	
342252	European handball (team) - moderate effort	8.0	Ainsworth 15330 - handball, team	[3]
342253	European handball (team) - hard effort	10.0	1.25 × moderate value	
321870	fishing	3.0	Ainsworth 04040 - fishing, general	[3]
**342150**	**football (Australian/American)**			
**342151**	**football (Australian/American) - light effort**	**6.6**	0.75 × moderate value	
**342152**	**football (Australian/American) - moderate effort**	**8.8**[a]	movement pattern similar to soccer measured in 1 child study, n = 25	[27]
**342153**	**football (Australian/American) - hard effort**	**11.0**	1.25 × moderate value	
342190	frisbee (general)			
342191	frisbee (general) - light effort	2.0	0.75 × moderate value	

342192	frisbee (general) - moderate effort	3.0	Ainsworth 15240 - frisbee playing, general	[3]
342193	frisbee (general) - hard effort	6.0	1.25 × moderate value	
342200	frisbee (ultimate)			
342201	frisbee (ultimate) - light effort	6.0	0.75 × moderate value	
342202	frisbee (ultimate) - moderate effort	8.0	Ainsworth 15250 - frisbee, ultimate	[3]
342203	frisbee (ultimate) - hard effort	10.0	1.25 × moderate value	
341210	golf			
341211	golf - light effort	3.0	Ainsworth 15270 - golf, miniature, driving ranges	[3]
341212	golf - moderate effort	4.3	Ainsworth 15285 - golf, walking and pulling clubs	[3]
341213	golf - hard effort	4.5	Ainsworth 15255 - golf, general	[3]
**341220**	**gymnastics**			
**341221**	**gymnastics - light effort**	**3.0**	0.75 × moderate value	
**341222**	**gymnastics - moderate effort**	**4.0**	gymnastics measured in 1 child study, n = 78.	[27]
**341223**	**gymnastics - hard effort**	**5.0**	1.25 × moderate value	
341230	hacky sack			
341231	hacky sack - light effort	3.0	0.75 × moderate value	
341232	hacky sack - moderate effort	4.0	Ainsworth 15310 - hacky sack	[3]
341233	hacky sack - hard effort	5.0	1.25 × moderate value	
**342240**	**hand tennis (four-square)**			
**342241**	**hand tennis (four-square) - light effort**	**3.8**	0.75 × moderate value	
**342242**	**hand tennis (four-square) - moderate effort**	**5.0**	playground games measured in 1 child study, n = 37	[27]
**342243**	**hand tennis (four-square) - hard effort**	**6.3**	1.25 × moderate value	
342830	hide and seek	4.0	Ainsworth 05175 - walk/run - playing with children, moderate	[3]
342260	hockey (field)			
342261	hockey (field) - light effort	6.0	0.75 × moderate value	

342262	hockey (field) - moderate effort	8.0	Ainsworth 15350 – field hockey	[3]
342263	hockey (field) - hard effort	10.0	1.25 × moderate value	
342270	hockey (ice)			
342271	hockey (ice) - light effort	6.0	0.75 × moderate value	
342272	hockey (ice) - moderate effort	8.0	Ainsworth 15360 - ice hockey	[3]
342273	hockey (ice) - hard effort	10.0	1.25 × moderate value	
**341280**	**hopscotch**			
**341281**	**hopscotch - light effort**	**4.4**	0.75 × moderate value	
**341282**	**hopscotch - moderate effort**	**5.9**	hopscotch measured in two child studies, n = 51	[14, 15]
**341283**	**hopscotch - hard effort**	**7.4**	1.25 × moderate value	
321290	horseback riding			
321291	horseback riding - light effort	2.5	Ainsworth 15400 - horseback riding, walking	[3]
321292	horseback riding - moderate effort	4.0	Ainsworth 15370 - horseback riding, general	[3]
321293	horseback riding - hard effort	6.5	Ainsworth 15390 - horseback riding, trotting	[3]
341300	ice skating			
341301	ice skating - light effort	5.5	Ainsworth 19020 - ice skating, 4 $m.s^{-1}$	[3]
341302	ice skating - moderate effort	7.0	Ainsworth 19030 - ice skating, general	[3]
341303	ice skating - hard effort	9.0	Ainsworth 19040 - ice skating, > 4 $m.s^{-1}$	[3]
331330	juggling	4.0	Ainsworth 15440 - juggling	[3]
341320	karate/martial arts/judo/kick boxing			
341321	karate/martial arts/judo/kick boxing - light effort	7.5	0.75 × moderate value	
341322	karate/martial arts/judo /kick boxing - moderate effort	10.0	Ainsworth 15430 - judo, jujitsu, karate, kick boxing, tae kwan do	[3]
341323	karate/martial arts/judo/kick boxing - hard effort	12.5	1.25 × moderate value	

341340	kayaking			
341341	kayaking - light effort	3.8	0.75 × moderate value	
341342	kayaking - moderate effort	5.0	Ainsworth 18100 - kayaking	[3]
341343	kayaking - hard effort	6.3	1.25 × moderate value	
342350	kickball			
342351	kickball - light effort	5.3	0.75 × moderate value	
342352	kickball - moderate effort	7.0	Ainsworth 15450 - kickball	[3]
342353	kickball - hard effort	8.8	1.25 × moderate value	
**342360**	**lacrosse**			
**342361**	**lacrosse - light effort**	**4.8**	0.75 × moderate value	
**342362**	**lacrosse - moderate effort**	**6.4**	lacrosse measured in 1 child study, n = 37	[27]
**342363**	**lacrosse - hard effort**	**8.0**	1.25 × moderate value	
342370	lawn bowls	3.0	Ainsworth 15570 - lawn bowling	[3]
**331960**	**lifting weights**			
**331961**	**lifting weights - light effort**	**2.1**	0.75 × moderate value	
**331962**	**lifting weights - moderate effort**	**2.8**	weight lifting (bench press and leg press) measured in 1 child study, n = 510	[8]
**331963**	**lifting weights - hard effort**	**3.5**	1.25 x moderate value	
341970	mini golf or putt putt	3.0	Ainsworth 15270 - golf, miniature, driving range	[3]
341980	unstructured indoor play - walk/run	4.0	Ainsworth 05175 - walk/run, playing with child(ren), moderate only active periods	[3]
321960	unstructured indoor play - sitting	2.3	Ainsworth 05170 - sitting, playing with child(ren): light, only active periods	[3]
**341990**	**unstructured outdoor play**			
**341991**	**unstructured outdoor play - light effort**	**3.8**	0.75 x moderate value	
**341992**	**unstructured outdoor play - moderate effort**	**5.0**	playground games measured in 1 child study, n = 37	[27]

**341993**	**unstructured outdoor play - hard effort**	**6.3**	1.25 × moderate value	
**342380**	**netball**			
**342381**	**netball - light effort**	**7.2**[a]	movement pattern similar to shooting hoops measured in 1 child study, n = 74	[35]
**342382**	**netball - moderate effort**	**8.2**[a]	movement pattern similar to basketball measured in 1 child study, n = 12	[37]
**342383**	**netball - hard effort**	**10.1**[a]	movement pattern similar to basketball – fast dribbling and shooting, measured in 1 child study, n = 19.	[26]
341390	orienteering			
341391	orienteering - light effort	7.0	0.75 × moderate value	
341392	orienteering - moderate effort	9.0	Ainsworth 15480 - orienteering	[3]
341393	orienteering - hard effort	11.0	1.25 × moderate value	
342400	paddleball			
342401	paddleball - light effort	4.5	0.75 × moderate value	
342402	paddleball - moderate effort	6.0	Ainsworth 15500 - paddleball	[3]
342403	paddleball - hard effort	7.5	1.25 × moderate value	
**341900**	**playground equipment (e.g. monkey bars)**			
**341901**	**playground equipment (e.g. monkey bars) - light effort**	**3.8**	0.75 × moderate value	
**341902**	**playground equipment (e.g. monkey bars) - moderate effort**	**5.0**	playground games measured in 1 child study, n = 37	[27]
**341903**	**playground equipment (e.g. monkey bars) - hard effort**	**6.3**	1.25 × moderate value	
**342770**	**playing catch**			
**342771**	**playing catch - light effort**	**2.0**	0.75 × moderate value	
**342772**	**playing catch - moderate effort**	**2.6**	catching measured in 2 child studies, n = 51	[14, 15]
**342773**	**playing catch - hard effort**	**3.3**	1.25 × moderate value	

**321880**	**playing in sandpit**	**1.6[a]**	movement pattern similar to sitting, playing puzzles measured in 3 child study, n = 312	[8, 23, 42]
321950	playing with animals - sitting	2.5	Ainsworth 05190 - sitting, playing with animals	[3]
341290	playing with animals - walk/run			
341291	playing with animals - walk/run - light effort	2.8	Ainsworth 05192 - walk/run, playing with animals, light	[3]
341292	playing with animals - walk/run - moderate effort	4.0	Ainsworth 05193 - walk/run, playing with animals, moderate	[3]
341293	playing with animals - walk/run - hard effort	5.0	Ainsworth 05194 - walk/run, playing with animals, vigorous	[3]
**321920**	**playing with toys/lego/dolls/action figures**	**1.6[a]**	movement pattern similar to sitting, playing puzzles measured in 3 child study, n = 312	[8, 23, 42]
342760	playing with young children			
342761	playing with young children - light effort	2.8	Ainsworth 05171 - standing, playing with children, light	[3]
342762	playing with young children - moderate effort	4.0	Ainsworth 05175 - walk/run - playing with children, moderate	[3]
342763	playing with young children - hard effort	5.0	Ainsworth 05180 - walk/run, playing with children, vigorous	[3]
**341820**	**pogo stick**			
**341821**	**pogo stick - light effort**	6.5	0.75 × moderate value	
**341822**	**pogo stick - moderate effort**	**8.7**	movement pattern similar to trampoline measured in 1 child study, n = 19.	[26]
**341823**	**pogo stick - hard effort**	10.9	1.25 × moderate value	
332040	pool/billiards/snooker	2.5	Ainsworth 15080 - billiards	[3]
331420	quoits	3.0	Ainsworth 15410 - horse pitching, quoits	[3]
341890	race walking			
341891	race walking - light effort	4.9	0.75 × moderate value	

341892	race walking - moderate effort	6.5	Ainsworth 17110 - race walking	[3]
341893	race walking - hard effort	8.1	1.25 × moderate value	
342430	racquetball			
342431	racquetball - light effort	5.3	0.75 × moderate value	
342432	racquetball - moderate effort	7.0	Ainsworth 15530 - racquetball, general	[3]
342433	racquetball - hard effort	8.8	1.25 × moderate value	
**342440**	**red rover/octopus tag/British bulldog/bullrush**			
**342441**	**red rover/octopus tag/British bulldog/bullrush - light effort**	**3.8**	0.75 × moderate value	
**342442**	**red rover/octopus tag/British bulldog/bullrush - moderate effort**	**5.0**	playground games measured in 1 child study, n = 37	[27]
**342443**	**red rover/octopus tag/British bulldog/bullrush - hard effort**	**6.3**	1.25 × moderate value	
**341240**	**riding a bicycle/bike**			
**341241**	**riding a bicycle/bike - light effort**	**4.7**	0.75 × moderate value	
**341242**	**riding a bicycle/bike - moderate effort**	**6.2**	riding a bicycle outdoors at 5.3 $m.s^{-1}$ measured in 1 child study, n = 74.	[35]
**341243**	**riding a bicycle/bike - hard effort**	**7.8**	1.25 × moderate value	
**341250**	**riding a scooter**			
**341251**	**riding a scooter - light effort**	**4.9**	0.75 × moderate value	
**341252**	**riding a scooter - moderate effort**	**6.5**	riding a scooter measured in 1 child study, n = 30	[39]
**341253**	**riding a scooter - hard effort**	**8.1**	1.25 × moderate value	
341270	riding a skateboard			
341271	riding a skateboard - light effort	3.8	0.75 × moderate value	
341272	riding a skateboard - moderate effort	5.0	Ainsworth 15580 - skateboarding	[3]
341273	riding a skateboard - hard effort	6.3	1.25 × moderate value	
341450	rockclimbing			
341451	rockclimbing - light effort	6.0	0.75 × moderate value	

341452	rockclimbing - moderate effort	8.0	Ainsworth 15540 - rock climbing, rapelling	[3]
341453	rockclimbing - hard effort	11.0	Ainsworth 15535 - rock climbing, ascending rock	[3]
**341310**	**rollerblading (in-line skating)**			
**341311**	**rollerblading (in-line skating) - light effort**	**4.9**	0.75 × moderate value	
**341312**	**rollerblading (in-line skating) - moderate effort**	**6.5**	rollerblading measured in 1 child study, n = 26	[39]
**341313**	**rollerblading (in-line skating) - hard effort**	**8.1**	1.25 × moderate value	
**341460**	**rollerskating**			
**341461**	**rollerskating - light effort**	**4.9**[a]	0.75 × moderate value	
**341462**	**rollerskating - moderate effort**	**6.5**[a]	movement pattern similar to rollerblading measured in 1 child study, n = 26	[39]
**341463**	**rollerskating - hard effort**	**8.1**[a]	1.25 × moderate value	
341090	rowing/canoeing			
341091	rowing/canoeing - light effort	3.0	Ainsworth (canoeing, rowing 2.0-3.9 mph, light effort)	[3]
341092	rowing/canoeing - moderate effort	7.0	Ainsworth 18050 (canoeing, rowing 4.0-5.9 mph, mod effort)	[3]
341093	rowing/canoeing - hard effort	12.0	Ainsworth 18060 (canoeing, rowing >6 mph, vig effort)	[3]
**342160**	**rugby league**			
**342161**	**rugby league - light effort**	**6.6**	0.75 × moderate value	
**342162**	**rugby league - moderate effort**	**8.8**[a]	movement pattern similar to soccer measured in 1 child study, n = 25	[27]
**342163**	**rugby league - hard effort**	**11.0**	1.25 × moderate value	
**342170**	**rugby union**			
**342171**	**rugby union - light effort**	**6.6**	0.75 × moderate value	
**342172**	**rugby union - moderate effort**	**8.8**[a]	movement pattern similar to soccer measured in 1 child study, n = 25	[27]

**342173**	**rugby union - hard effort**	**11.0**	1.25 × moderate value	
**341480**	**running /jogging**			
**341481**	**running/jogging - light effort**	**7.7[b]**	regression equation developed based on 31 child studies: METs = 0.27 age + 1.91 speed ($m.s^{-1}$) + 0.46, r = 0.61, SEE = 1.4 METs, n = 1974. Light speed[d] = 2.08 $m.s^{-1}$	[9]
**341482**	**running/jogging - moderate effort**	**8.5[b]**	As above Moderate speed[d] = 2.50 $m.s^{-1}$	[9]
**341483**	**running/jogging - hard effort**	**9.3[b]**	As above Hard speed[d] = 2.92 $m.s^{-1}$	[9]
331490	sailboard/windsurfing			
331491	sailboard/windsurfing - light effort	2.0	0.75 × moderate value	
331492	sailboard/windsurfing - moderate effort	3.0	Ainsworth 18120 - windsurfing	[3]
331493	sailboard/windsurfing - hard effort	5.0	1.25 × moderate value	
321940	sailing/boating			
321941	sailing/boating - light effort	2.3	0.75 × moderate value	
321942	sailing/boating - moderate effort	3.0	Ainsworth 18120 - sailing, boat sailing, wind surfing	[3]
321943	sailing/boating - hard effort	3.8	1.25 × moderate value	
341500	shuffleboard			
341501	shuffleboard - light effort	2.3	0.75 × moderate value	
341502	shuffleboard - moderate effort	3.0	Ainsworth 15570 - shuffleboard, lawn bowling	[3]
341503	shuffleboard - hard effort	4.0	1.25 × moderate value	
341520	skiing (cross-country)			
341521	skiing (cross-country) - light effort	7.0	Ainsworth 19080 - cross country skiing, slow or light effort	[3]
341522	skiing (cross-country) - moderate effort	8.0	Ainsworth 19090 - cross country skiing, moderate speed and effort	[3]
341523	skiing (cross-country) - hard effort	9.0	Ainsworth 19100 - cross country skiing, fast effort	[3]

341530	skiing (downhill)			
341531	skiing (downhill) - light effort	5.0	Ainsworth 19150 - skiing, downhill, light effort	[3]
341532	skiing (downhill) - moderate effort	6.0	Ainsworth 19160 - skiing, downhill, moderate effort	[3]
341533	skiing (downhill) - hard effort	8.0	Ainsworth 19170 - skiing, downhill, vigorous effort	[3]
341540	skindiving (SCUBA)			
341541	skindiving (SCUBA) - light effort	7.0	Ainsworth 18200 - skindiving, scuba diving, general	[3]
341542	skindiving (SCUBA) - moderate effort	12.5	Ainsworth 18190 - skindiving, moderate	[3]
341543	skindiving (SCUBA) - hard effort	16.0	Ainsworth 18180 - skindiving - fast	[3]
**341470**	**skipping/jump rope**			
**341471**	**skipping/jump rope - light effort**	**6.2**	0.75 × moderate value	
**341472**	**skipping/jump rope - moderate effort**	**8.3**	skipping measured in 1 child study, n = 262	[8]
**341473**	**skipping/jump rope - hard effort**	**10.3**	1.25 × moderate value	
341550	snorkelling			
341551	snorkelling - light effort	3.8	0.75 × moderate value	
341552	snorkelling - moderate effort	5.0	Ainsworth 18210 - snorkeling	[3]
341553	snorkelling - hard effort	6.3	1.25 × moderate value	
**342180**	**soccer (field/indoor)**			
**342181**	**soccer (field/indoor) - light effort**	**6.6**	0.75 × moderate value	
**342182**	**soccer (field/indoor) - moderate effort**	**8.8**	soccer measured in 1 child study, n = 25	[27]
**342183**	**soccer (field/indoor) - hard effort**	**11.0**	1.25 × moderate value	
342560	softball or t-ball			
342561	softball or t-ball - light effort	3.8	0.75 × moderate value	
342562	softball or t-ball - moderate effort	5.0	Ainsworth 15620 - baseball or softball, general	[3]
342563	softball or t-ball - hard effort	6.3	1.25 × moderate value	
341570	speed skating (competitive)			

341571	speed skating (competitive) - light effort	7.0	Ainsworth 19030 - ice skating, general	[3]
341572	speed skating (competitive) - moderate effort	9.0	Ainsworth 19040 - ice skating, > 4 m.s-1	[3]
341573	speed skating (competitive) - hard effort	15.0	Ainsworth 19050 - speed skating, competitive	[3]
342580	squash			
342581	squash - light effort	5.3	0.75 × moderate value	
342582	squash - moderate effort	7.0	movement pattern similar to racquetball - Ainsworth 15530	[3]
342583	squash - hard effort	8.8	0.75 × moderate value	
**331590**	**stretching exercises**	**2.5**	stretching exercises measured in 1 child study, n = 264	[8]
341600	surfing (body or board)			
341601	surfing (body or board) - light effort	3.8	0.75 × moderate value	
641602	surfing (body or board) - moderate effort	5.0	Ainsworth 18220 - surfing, body or board	[3]
341603	surfing (body or board) - hard effort	6.3	1.25 × moderate value	
341930	swimming (playing in pool)			
341931	swimming (playing in pool) - light effort	3.0	Ainsworth 18365 - water volleyball	[3]
341932	swimming (playing in pool) - moderate effort	4.0	Ainsworth 18350 - swimming, treading water moderate effort	[3]
341933	swimming (playing in pool) - hard effort	5.0	1.25 × moderate value	
**341610**	**swimming laps**			
**341611**	**swimming laps - light effort**	**8.4**	swimming at 0.9 $m.s^{-1}$ measured in 1 child study, n = 11	[40]
**341612**	**swimming laps - moderate effort**	**9.9**	swimming at 1.0 $m.s^{-1}$ measured in 1 child study, n = 21	[40]
**341613**	**swimming laps - hard effort**	**11.6**	swimming at 1.1 $m.s^{-1}$ measured in 1 child study, n = 11	[40]
342620	table tennis			

342621	table tennis - light effort	3.0	0.75 × moderate value	
342622	table tennis - moderate effort	4.0	Ainsworth 15660 - table tennis, ping pong	[3]
342623	table tennis - hard effort	5.0	1.25 × moderate value	
**331630**	**tai chi/yoga**	**2.5**[a]	stretching exercises measured in 1 child study, n = 264	[8]
**341800**	**tap dancing**			
**341801**	**tap dancing - light effort**	**3.0**	0.75 × moderate value	
**341802**	**tap dancing - moderate effort**	**4.8**	dancing measured in one child study, n = 36	[27]
**341803**	**tap dancing - hard effort**	**7.0**	1.25 × moderate value	
342640	tennis (court)			
342641	tennis (court) - light effort	5.3	0.75 × moderate value	
342642	tennis (court) - moderate effort	7.0	Ainsworth 15675 - tennis, general	[3]
342643	tennis (court) - hard effort	8.8	1.25 × moderate value	
**342060**	**tenpin bowling**	**4.9**	bowling measured in 1 child study, n = 12	[37]
331990	totem tennis			
331991	totem tennis - light effort	3.8	0.75 × moderate value	
331992	totem tennis - moderate effort	5.0	Ainsworth 15135 - children's games, tetherball	[3]
331993	totem tennis - hard effort	6.3	1.25 × moderate value	
**342810**	**touch football**			
**342811**	**touch football - light effort**	**6.6**	0.75 × moderate value	
**342812**	**touch football - moderate effort**	**8.8**[a]	movement pattern similar to soccer measured in 1 child study, n = 25	[27]
**342813**	**touch football - hard effort**	**11.0**[a]	1.25 × moderate value	
**341680**	**trampoline**			
**341681**	**trampoline - light effort**	6.5	0.75 × moderate value	
**341682**	**trampoline - moderate effort**	8.7	trampoline measured in 1 child study, n = 19.	[26]
**341683**	**trampoline - hard effort**	10.9	1.25 × moderate value	
342700	volleyball (beach)			

342701	volleyball (beach) - light effort	6.0	0.75 × moderate value	
342702	volleyball (beach) - moderate effort	8.0	Ainsworth 15725 - beach volleyball	[3]
342703	volleyball (beach) - hard effort	10.0	1.25 × moderate value	
342690	volleyball (court)			
342691	volleyball (court) - light effort	3.0	0.75 × moderate value	
342692	volleyball (court) - moderate effort	4.0	Ainsworth 15710 - volleyball	[3]
342693	volleyball (court) - hard effort	5.0	1.25 × moderate value	
342710	wallyball/modified volleyball			
342711	wallyball/modified volleyball - light effort	5.3	0.75 × moderate value	
342712	wallyball/modified volleyball - moderate effort	7.0	Ainsworth 15731 - wallyball, general	[3]
342713	wallyball/modified volleyball - hard effort	8.8	1.25 × moderate value	
331730	water skiing			
331731	water skiing - light effort	4.5	0.75 × moderate value	
331732	water skiing - moderate effort	6.0	Ainsworth 18150 - skiing, water	[3]
331733	water skiing - hard effort	7.5	1.25 × moderate value	
341740	whitewater rafting			
341741	whitewater rafting - light effort	3.8	0.75 × moderate value	
341742	whitewater rafting - moderate effort	5.0	Ainsworth 18370 - whitewater rafting, kayaking, canoeing	[3]
341743	whitewater rafting - hard effort	6.3	1.25 × moderate value	
342860	wrestling with mates	4.0	Ainsworth 05175 - walk/run, playing with child(ren), moderate only active periods	[3]
	**SCHOOL WORK**			
**420000**	**arts and crafts - sitting**	**1.6**	arts and crafts, sitting measured in 2 child studies, n = 101	[27, 36]
**430000**	**arts and craft - standing**	**1.9**	drawing, standing measured in 1 child study, n = 19	[38]

**440160**	**carrying sports equipment**	**4.2[a]**	walking carrying an 8.5 kg backpack measured in 1 child study, n = 35	[32]
420050	computer work (e.g. typing/internet)	1.8	Ainsworth 09040 - sitting, typing	[3]
**420090**	**crayoning**	**1.4**	crayoning measured in 2 child studies, n = 51	[14, 15]
430070	drama class	2.3	structure of drama class assumed to be 0.5 schoolwork (e.g. sitting, listening) and 0.5 acting (theatre actor)	[3]
**420010**	**drawing - sitting**	**1.4[a]**	movement pattern similar to crayoning measured in 2 child studies, n = 51	[14, 15]
**430100**	**drawing - standing**	**1.9**	drawing, standing measured in 1 child study, n = 19	[38]
**430080**	**giving a talk/presentation**	**1.6[a]**	movement pattern similar to standing, singing measured in 1 child study, n = 17	[20]
**420130**	**morning organisation/books and pens out**	**1.4**	school work, sitting measured in 5 studies, n = 442	[8, 23, 27, 29, 36]
440170	packing up/chairs up - end of school	2.5	movement pattern similar to Ainsworth 05040 – cleaning, light (dusting, straightening up, changing linen, carrying out trash)	[3]
430140	packing/unpacking bag	2.5	Ainsworth 05055 - home activities, putting away groceries	[3]
420020	reading - sitting	1.3[a]	Ainsworth 09030 - sitting - reading book, newspaper, etc	[3]
430020	reading - standing	1.8	Ainsworth 09070 - standing reading	[3]
**420120**	**roll call/class organisation**	**1.4**	sitting quietly measured in 9 child studies, n = 288	[19, 21, 22, 25, 27-31]
430150	school crossing monitor	2.0	Ainsworth 09071 - standing, miscellaneous	[3]

420180	science experiments – sitting	1.5	Ainsworth 11580 – sitting, chemistry lab work	[3]
430190	science experiments – standing	2.5	Ainsworth 11430 – machine tooling	[3]
**420110**	**sitting quietly (e.g. assembly/listening to teacher)**	**1.4**	sitting quietly measured in 9 child studies, n = 288	[19, 21, 22, 25, 27-31]
**420030**	**studying/homework**	**1.4**	school work, sitting measured in 5 studies, n = 442	[8, 23, 27, 29, 36]
**420040**	**taking notes/class discussion**	**1.4**	school work, sitting measured in 5 studies, n = 442	[8, 23, 27, 29, 36]
430090	technical studies - standing light effort (e.g. electronics/machining etc)	2.5	Ainsworth 11430 – machine tooling	[3]
430110	technical studies - standing moderate effort (e.g. carpentry/welding)	3.0	Ainsworth 06040 – carpentry, general	[3]
**420060**	**writing - sitting**	**1.4**	school work, sitting measured in 5 studies, n = 442	[8, 23, 27, 29, 36]
	**SELF CARE**			
531000	brushing or combing hair (standing)	2.5	Ainsworth 13045 - hairstyling	[3]
531010	brushing teeth	2.0	Ainsworth 13040 - grooming, brushing teeth	[3]
**533020**	**dressing & undressing**	**2.7**	dressing and undressing measured in 2 child studies, n = 93	[27, 38]
522130	drinking - sitting	1.5	movement pattern similar to Ainsworth 13030 - eating, sitting	[3]
532140	drinking while standing	2.0	movement pattern similar to Ainsworth 13035 - eating, standing	[3]
522030	eating - sitting	1.5	Ainsworth 13030 - eating, sitting	[3]
532040	eating while standing	2.0	Ainsworth 13035 - eating, standing	[3]
**553050**	**getting ready for bed**	**2.7**	movement pattern is dressing and undressing measured in 2 child studies, n = 93	[27, 38]

**521060**	**having hair done by someone else**	**1.4[a]**	sitting quietly measured in 9 child studies, n = 288	[19, 21, 22, 25, 27-31]
534120	putting on makeup/sunscreen/contact lenses/grooming etc	2.0	Ainsworth 13040 - grooming, putting on make-up	[3]
531070	showering & towelling off	2.0	Ainsworth 13050 - showering, towelling off	[3]
**521080**	**sitting in bath**	**1.4[a]**	sitting quietly measured in 9 child studies, n = 288	[19, 21, 22, 25, 27-31]
**521090**	**sitting on toilet**	**1.4[a]**	sitting quietly measured in 9 child studies, n = 288	[19, 21, 22, 25, 27-31]
531100	taking medicine	1.5	Ainsworth 13030 - eating, sitting	[3]
534110	washing hands/face	2.0	Ainsworth 13040 - grooming, washing	[3]
	**CHORES**			
**632000**	**bathing dog**	**2.5**	caring for animals measured in 1 child study, n = 17	[27]
642010	carrying small children	3.0	Ainsworth 05181 - carrying small children	[3]
641410	carrying very heavy items (e.g. moving furniture)	6.0	Ainsworth 05120 – carrying very heavy furniture	[3]
622020	child care (e.g. dressing)	3.0	Ainsworth 05186 - child care standing, dressing	[3]
622030	child care (e.g. feeding)	2.5	Ainsworth 05185 - child care sitting/kneeling, feeding	[3]
633400	chopping wood	6.0	Ainsworth 08020 – chopping wood	[3]
630040	cooking – standing	2.0	Ainsworth 05050- cooking or food preparation	[3]
**632050**	**doing the laundry**	**2.6[a]**	hanging out washing measured in 1 child study, n = 14	[39]
**641060**	**dusting**	**4.2**	dusting measured in 1 child study, n = 24	[23]
**642070**	**feeding pets or farm animals**	**2.5**	caring for animals measured in 1 child study, n = 17	[27]

642360	fixing things up (e.g. bike/toys/mechanical work on car etc)	2.5	Ainsworth 05025, multiple household tasks all at once, light effort	[3]
**643330**	**gardening**	**3.7**	gardening measured in 1 child study, n = 63	[27]
**642080**	**hanging out washing**	**2.6**	hanging out washing measured in 1 child study, n = 14	[39]
632090	ironing	2.3	Ainsworth 05070 - ironing	[3]
622100	knitting or sewing	1.5	Ainsworth 05080 - sitting, knitting, sewing	[3]
630370	making recess or lunch	2.0	Ainsworth 05050 - cooking or food preparation	[3]
**631110**	**making the bed**	**3.4**	making bed measured in 2 child studies, n = 78	[27, 39]
**641120**	**mopping**	**3.6**[a]	sweeping measured in 4 child studies, n = 378	[8, 23, 35, 37]
643290	mowing lawn	5.5	Ainsworth 08120 - mowing lawn, walk, power mower	[3]
633340	picking flowers fruit or vegetables	3.0	Ainsworth 08246 - picking fruit/vegetables	[3]
**623320**	**pulling up weeds**	**3.7**	gardening measured in 1 child study, n = 63	[27]
641130	putting away clothes	2.3	Ainsworth 05095 - putting away clothes, implied walking	[3]
642170	putting away or carrying groceries	2.5	Ainsworth 05055 - putting away groceries	[3]
643310	raking leaves	4.3	Ainsworth 08160 - raking lawn	[3]
641180	scrubbing floors in bathroom or bathtub	3.8	Ainsworth 05130 - scrubbing floors	[3]
**640190**	**serving food**	**2.6**	setting table measured in 1 child study, n = 20	[39]
**640350**	**setting the table**	**2.6**	setting table measured in 1 child study, n = 20	[39]
642200	shopping	2.3	Ainsworth 05060 - food shopping	[3]
**633390**	**shoveling/digging**	**4.3**	shoveling measured in 1 child study, n = 285	[8]

**641210**	**sweeping floor**	**3.6**	sweeping measured in 4 child studies, n = 378	[8, 23, 35, 37]
**643220**	**sweeping outside**	**3.6**	sweeping measured in 4 child studies, n = 378	[8, 23, 35, 37]
641370	taking out the rubbish/garbage bin	2.5	Ainsworth 05030 - cleaning, light, carrying out trash	[3]
**641230**	**tidying/cleaning room**	**3.4**	making bed and cleaning room measured in 2 child studies, n = 78	[27, 39]
631240	unpacking boxes	3.5	Ainsworth 05146 - standing - unpacking, packing boxes	[3]
**641250**	**vacuuming**	**4.2**	vacuuming measured in 3 child studies, n = 299	[8, 23, 29]
642260	washing car or windows	3.0	Ainsworth 05020 - cleaning, heavy, washing car or windows	[3]
**630270**	**washing or clearing dishes**	**1.9**	washing dishes measured in 2 child studies, n = 32	[29, 39]
643280	watering plants	2.5	Ainsworth 05148 - watering plants	[3]
643300	wheelbarrowing	5.0	Ainsworth 08010 - loading/unloading or carrying lumber	[3]
	**OTHER**			
721030	amusement/theme park rides	1.5	movement pattern similar to Ainsworth 09115 - watching sporting events	[3]
721000	camping – sitting	2.5	Ainsworth 09110 - camping involving standing, walking, sitting	[3]
**722190**	**computer/playstation games**	**1.5**	playing video games sitting measured in 4 child studies, n = 426	[8,23,36,41]
**732200**	**playing active video games (e.g. EyeToy/dance mat/arcade games)**			
**732201**	**playing active video games (e.g. EyeToy/dance mat/arcade games) - light effort**	**1.7**	playing video centre games measured in 2 child studies, n = 303	[8, 33]

**732202**	**playing active video games (e.g. EyeToy/dance mat/arcade games) - moderate effort**	**3.4**	playing moderate intensity video centre games (e.g. air hockey) measured in 1 child study, n = 10	[41]
**732203**	**playing active video games (e.g. EyeToy/dance mat/arcade games) - hard effort**	**6.4**	playing high intensity video games (e.g. simulated horse racing) measured in 1 child study, n = 10	[41]
731210	darts	2.5	Ainsworth 15180 - darts, wall or lawn	[3]
721010	family get-togethers (eat talk sit relax)	1.5	Ainsworth 09000 - retreat/family reunion activities involving sitting, relaxing, talking, eating	[3]
723020	part time work - child care sitting (e.g. feeding/bathing)	2.5	Ainsworth 05185 – child care, general	[3]
733030	part time work - child care standing (e.g. carrying/dressing/playing)	3.0	Ainsworth 05186 – child care	[3]
**743020**	**part time work - door to door sales/delivery - paper rounds etc (long periods of walking)**	**2.9**[a]	based on walking – light (see code 240051)	
**743030**	**part time work - door to door sales/delivery - paper rounds etc (riding bike)**	**4.7**[a]	based on riding a bicycle – light (see code 341241)	
733010	part time work - serving standing (e.g. checkout/fast food)	2.3	Ainsworth 11600 – standing light (bartending, store clerk, etc)	[3]
723010	part time work - sitting office work	1.5	Ainsworth 11580 – sitting, light office work	[3]
733020	part time work - stacking shelves	3.0	Ainsworth 11610 – standing; light/moderate (stocking)	[3]
743010	part time work - waitressing etc (short periods of walking)	2.0	Ainsworth 11791 – walking on job, very slow	[3]
**741260**	**partying (mostly dancing)**	**3.2**[a]	based on 60% light dancing (4.1 METs) & 40% standing talking (1.8 METs).	
721240	partying (mostly sitting)	1.5	Ainsworth 09055 - sitting talking, or talking on the phone	[3]

731250	partying (mostly standing)	1.8	Ainsworth 09050 - standing talking or talking on the phone	[3]
740150	performing on stage at assembly	3.0	Ainsworth 11870 - theatre actor	[3]
**721220**	**playing cards/puzzles/board games**	**1.6**	playing with puzzles measured in 3 child studies, n = 312	[8, 23, 42]
720030	playing the accordion	1.8	Ainsworth 10010 - music playing, accordion	[3]
**720040**	**playing the cello**	**1.7**	cello playing measured in 1 child study, n = 8	[34]
720050	playing the drums	4.0	Ainsworth 10040 - music playing, drums	[3]
720060	playing the flute/recorder - sitting	2.0	Ainsworth 10050 - music playing, flute (sitting)	[3]
720070	playing the guitar - sitting	2.0	Ainsworth 10120 - music playing, guitar (sitting)	[3]
730080	playing the guitar – standing	3.0	Ainsworth 10125 - music playing, guitar, standing	[3]
720090	playing the horn	2.0	Ainsworth 10060 - music playing, horn	[3]
**720100**	**playing the piano or organ**	**1.7**	playing the piano measured in 1 child study, n = 29	[27]
730110	playing the trombone	3.5	Ainsworth 10080 - music playing, trombone	[3]
730120	playing the trumpet	2.5	Ainsworth 10090 - trumpet	[3]
**720130**	**playing the violin**	**1.7**	violin playing measured in 2 child studies, n = 17	[27, 34]
720140	playing woodwind instruments	1.6	Ainsworth 10110 - music playing, woodwind instruments	[3]
**721150**	**praying**	**1.4**[a]	sitting quietly measured in 9 child studies, n = 288	[19, 21, 22, 25, 27-31]
**720180**	**singing – sitting**	**1.4**	singing, sitting measured in 1 child study, n = 15	[38]
**730180**	**singing – standing**	**1.6**	singing, standing measured in 1 child study, n = 17	[38]

**721160**	**sitting in church**	**1.5**	Ainsworth 20005 - sitting in church, talking or singing	[3]
721230	watching live sporting events	1.5	Ainsworth 09115 - watching sporting events	[3]
722170	wrapping/unwrapping presents	1.5	Ainsworth 05080 - wrapping presents	[3]

Note: MET = metabolic equivalent; TV = television; SEE **=** standard error of estimate; Adult = Adult compendium codes - code descriptions are not consistently presented in full. Some code descriptions have been edited to SI units and only relevant descriptions presented to conserve table space;

[a] = in some instances, the child specific energy cost for an activity with an identical movement pattern is used rather than an adult Ainsworth value with a more similar activity description;

[b] = walking and running energy costs based on a 12 year old child;

[c] = light, moderate and hard effort walking speeds based on speeds commonly reported in the Ridley and Olds energy cost review [9] and the customary slow, normal and fast walking speeds of 6-19 year olds reported by Waters and colleagues [16];

[d] = light, moderate and hard effort running speeds based on speeds commonly reported in the Ridley and Olds energy cost review [9].
